# Sensitivity analysis of disease-information coupling propagation dynamics model parameters

**DOI:** 10.1371/journal.pone.0265273

**Published:** 2022-03-25

**Authors:** Yang Yang, Haiyan Liu

**Affiliations:** School of Economics and Management, China University of Geosciences (Beijing), Beijing, China; University of Bradford, UNITED KINGDOM

## Abstract

The disease-information coupling propagation dynamics model is a widely used model for studying the spread of infectious diseases in society, but the parameter settings and sensitivity are often overlooked, which leads to enlarged errors in the results. Exploring the influencing factors of the disease-information coupling propagation dynamics model and identifying the key parameters of the model will help us better understand its coupling mechanism and make accurate recommendations for controlling the spread of disease. In this paper, Sobol global sensitivity analysis algorithm is adopted to conduct global sensitivity analysis on 6 input parameters (different cross regional jump probabilities, information dissemination rate, information recovery rate, epidemic transmission rate, epidemic recovery rate, and the probability of taking preventive actions) of the disease-information coupling model with the same interaction radius and heterogeneous interaction radius. The results show that: (1) In the coupling model with the same interaction radius, the parameters that have the most obvious influence on the peak density of nodes in state *A*_*I*_ and the information dissemination scale of the information are the information dissemination rate *β*_*I*_ and the information recovery rate *μ*_*I*_. In the coupling model of heterogeneous interaction radius, the parameters that have the most obvious impact on the peak density of nodes in the *A*_*I*_ state of the information layer are: information spread rate *β*_*I*_, disease recovery rate *μ*_*E*_, and the parameter that has a significant impact on the scale of information spread is the information spread rate *β*_*I*_ and information recovery rate *μ*_*I*_. (2) Under the same interaction radius and heterogeneous interaction radius, the parameters that have the most obvious influence on peak density of nodes in state *S*_*E*_ and the disease transmission scale of the disease layer are the disease transmission rate *β*_*E*_, the disease recovery rate *μ*_*E*_, and the probability of an individual moving across regions *p*_*jump*_.

## 1. Introduction

At present, new coronary pneumonia is still raging around the world, and information about the disease will also spread on social networking platforms, and the spread of information may have an effect on or inhibit or promote the spread of the disease. These two types of spread are often show a coupling relationship. In our existing research result [1], we explored the influence of a single parameter on the model, but did not consider the influence of the interaction of the parameters on the model. In order to better understand the coupling mechanism of these two transmission processes and propose measures that can accurately control the spread of the disease, this paper conducts a sensitivity analysis on the parameters of the disease-information coupling transmission dynamics model, and uses quantitative methods to identify the important effects of the dynamics model factor.

Sensitivity analysis is the prescriptive or quantitative analysis of the effect of model inputs (including model parameters) on model outputs [2]. In general, one might be interested in which parameters have the greatest impact on the output, and which parameters have negligible impact [3]. Model parameter sensitivity analysis can diagnose the model structure and identify the key parameters of the model, which is a key step in model establishment and application [4]. Sensitivity analysis can be divided into local sensitivity analysis and global sensitivity analysis. Local sensitivity analysis is usually carried out by calculating partial derivatives by analytical or numerical methods, usually by perturbing one parameter at a time [2]. However, this method can only evaluate the influence of a single parameter on the model output, and cannot evaluate the influence of the interaction between parameters on the model output. Although it is easy to operate, it has great limitations due to the phenomenon of “same effect with different parameters”. Global sensitivity analysis is to analyze the common influence of multiple parameters on the model output and the interaction among parameters in the whole parameter space. It is more suitable for the research and analysis of complex systems.

The global sensitivity analysis method is developed on the basis of local sensitivity analysis. Compared with local sensitivity analysis, global sensitivity analysis on the one hand takes into account the influence of the distribution and shape of probability density function of each factor, and on the other hand, all factors can change simultaneously during calculation and analysis. Cukier et al. [5], Iman et al. [6], Archer et al. [7], Saltelli et al. [8] and other scholars have successively studied global sensitivity analysis methods. The characteristics of global sensitivity analysis are as follows: the range of factor variation can be extended to the entire definition domain of the factor; each factor allows different ranges of variation and can vary simultaneously; not limited by model, nonlinear, non-superposition and non-monotonic models can be studied.

The global sensitivity analysis methods are mainly as follows:

1. Screening method. This method is usually used to deal with models with a large number of input factors, and the amount of computation is relatively small. When there are many factors in the model, first use the screening method to determine the factors that have a greater impact on the model output, remove the factors that have little impact, and then use other methods for sensitivity analysis, which can greatly simplify the calculation. However, the screening method can only do qualitative analysis, and cannot give specific quantitative results of the importance of one parameter over another parameter. (2) Monte Carlo method. It is a numerical simulation method that constructs random variables by random sampling from the probability distribution of known model inputs. Then, according to the calculation results of random variables, the uncertain factors of the output are determined, and then they are apportioned to the uncertain factors in the input. (3) Variance-based methods. Cukier et al. [5] originally used the Fourier Amplitude Sensitivity Test (FAST) and then extended it as in [9]. The variance-based method can calculate the sensitivity index by decomposing the output variance into the first-order and higher-order effects of the input. Commonly used methods include important estimation method, Fourier method, Sobol method and so on. This method has unique advantages in sensitivity analysis due to its variance-based analysis.

Sensitivity analysis based on variance has made some progress and has been applied in many fields. Song et al. [10] used variance-based sensitivity method and GRSA method to conduct global sensitivity analysis on headless rivet model and Ten-bar structure model. Savolainen [3] used the Sobol method based on variance to conduct a global sensitivity analysis of feedback control stochastic process models, and discussed how to use global sensitivity analysis in dynamic and stochastic process modeling cases. Scholars such as Zhou [11] introduced the sparse grid integration method into the calculation of the sensitivity index based on variance and applied it to the automobile front axle model. The practical application shows that this method inherits the advantages of sparse grid integration in integral estimation, and controls the computation while ensuring the accuracy of sensitivity analysis. Scholars such as Fonoberova discussed the global sensitivity analysis based on the multi-agent model [12–18]. The Sobol method based on variance decomposition has been shown to be an appropriate sensitivity analysis method [3, 19].

The dynamic model of disease-information transmission (abbreviated: DMDT) is a widely used method. The effect of parameter sensitivity on the results is ignored, leading to deviations in the results. Therefore, how parameter changes affect the results of disease information dynamics models has become a problem that needs to be studied. Currently, there are relatively few sensitivity analyses for disease and information dissemination network models. This paper uses the widely used and representative Sobol method to analyze the global sensitivity of the disease-information coupling propagation dynamics model constructed in the published article [1]. At the same time, we further improved the model and proposed a disease-information coupling model based on heterogeneous interaction radius. The influence of each parameter and the interaction between the parameters on the two models is obtained by quantitative analysis. This study can complement the application of sensitivity analysis methods in the field of disease-information coupling transmission.

The rest of this article is as follows: Part 2 introduces the disease-information two-layer coupling model and coupling propagation dynamics model to be analyzed. Part 3 introduces the principle and calculation of Sobol global sensitivity analysis method. Part 4 is sensitivity analysis and simulation results and analysis of sensitive factors. Part 5 is the conclusion and outlook.

## 2. DMDT parameter selection

DMDT is based on the disease-information two-layer network model, which is divided into a disease layer and an information layer, representing social networks and physical contact networks, respectively. Information and disease spread in the information layer and disease layer respectively. In this double-layer network, nodes between layers are connected by dotted lines, indicating that these two nodes are the same individual. In this double-layer network model, we assume that the network structure of the information layer is static in the short term. This is because the social relationship between people is generally relatively stable in the short term and will not change much. The network structure of the disease layer is dynamically changing, because in real life people always move due to various factors such as work, life, travel, etc., and meet different people at different times, which causes the structure of the physical contact network to change from moment to moment. The transmission dynamics of the disease layer and the information layer use the SIR model. Individuals in the information layer have three states. State *U*_*I*_ means that individuals have not received epidemic-related information. State *A*_*I*_ represents that individuals have received epidemic-related information. State *N*_*I*_ means that individuals have received epidemic-related information but do not pass that information to others. Individuals in the disease layer also have three states. State *S*_*E*_ means individuals are not sick, but they can be infected by sick neighbors with a certain probability. State *I*_*E*_ means individuals are already sick, and will infect their neighbors with a certain probability. Individuals in state *R*_*E*_ are no longer infected and cannot infect other individuals. For detailed information of the model, please refer to [1].

**Fig 1:**
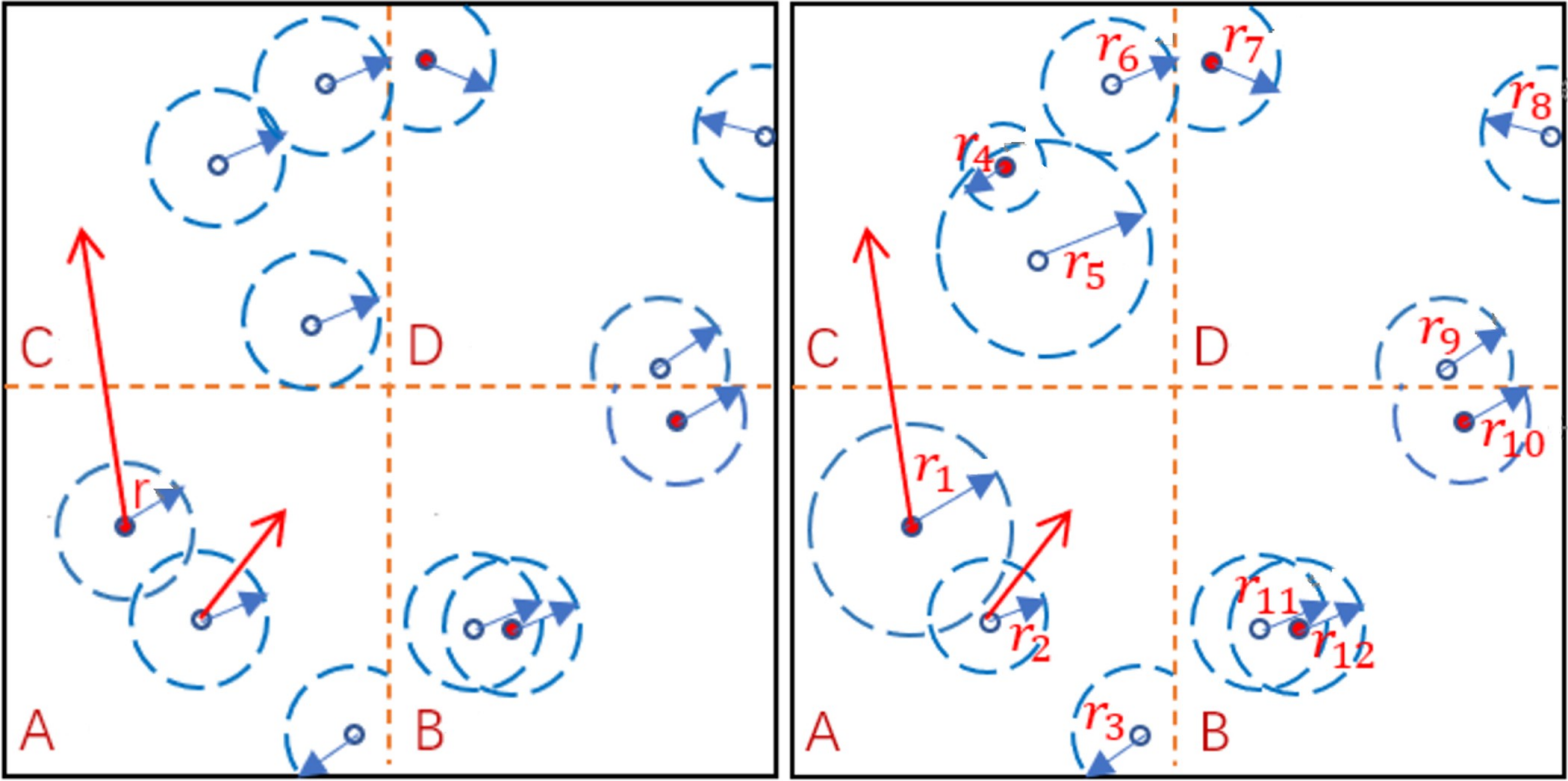
(a) Each individual has the same interaction radius. (b) Each individual has a heterogeneous interaction radius.

In the above model, we assume that each individual has the same interaction radius, as shown in Fig 1A. However, in reality, the individual interaction radius should be heterogeneous [20]. Considering the influence of the heterogeneity of the interaction radius between individuals on the spread of disease, we have further improved the above model. In the improved model, each individual *i* has its own radius, denoted by *r*_*i*_. Individual *i* can be infected by infected individuals within the radius of *r*_*i*_. As shown in Fig 1B below. For simplicity, we only give 12 individuals here, and the interaction radius of each individual is: *r*_1_, *r*_2_,…,*r*_12_. Individual 5 and individual 11 can be infected by individual 4 and individual 12, respectively, but individual 2 cannot be infected by individual 1. Since the interaction radius represents the neighborhood where a person may be infected by infected neighbors in the area, we also call it the susceptibility radius. We assume that, in our model, there are m different interaction radius values, which obey the distribution *P*(*r*_*j*_), *j* = 1,…,*m*, where *P*(*r*_*j*_) represents the node with the interaction radius *r*_*j*_ proportion. Referring to the existing research [20], here we set the distribution of *r* as: *r* = [0.5 1 1.5] and *P*(*r*) = [0.3 0.4 0.3].

The parameters involved in these two models, their meanings and value ranges are shown in Table 1.

**Table 1:** Model parameter description.

Parameter	Description of parameters	Value range
information dissemination rate *β*_*I*_	The probability of an individual changing from *U*_*I*_ state to *A*_*I*_ state	[0,1]
information recovery rate *μ*_*I*_	The probability of an individual changing from *A*_*I*_ state to *N*_*I*_ state	[0,1]
epidemic transmission rate *β*_*E*_	The probability of an individual changing from the *S*_*E*_ state to the *I*_*E*_ state	[0,1]
epidemic recovery rate *μ*_*E*_	The probability of an individual changing from *I*_*E*_ state to *R*_*E*_ state	[0,1]
The probability of taking preventive actions ω	Probability of individuals who are informed and not infected with the disease to take preventive behaviors	[0,1]
Different cross regional jump probabilities *p*_*jump*_	The probability of an individual moving from the current area to another area	[0,1]

In these two models, there are four output variables to be studied, namely: the peak density of nodes in the *A*_*I*_ state of the information layer, which we denote by $\rho_{A_{I}}$ here; the density of nodes in the *N*_*I*_ state when the information layer dissemination approaches the end, which represents the scale of information dissemination, here is represented by $\rho_{N_{I}}$; the peak density of nodes in the *S*_*E*_ state of the disease layer is represented by $\rho_{S_{E}}$; the density of nodes in the *R*_*E*_ state when the spread of the disease layer approaches the end, which represents the scale of disease transmission, here is represented by $\rho_{R_{E}}$.

## 3. Sensitivity analysis principle and calculation

### 3.1 Principle of Sobol method

The Sobol method is a quantitative global sensitivity analysis method based on variance. Its basic principles are as follows:

Given a square integrable function, the domain of the function is:

$$\Omega^{k}=(X|0\leq x_{i}\leq 1;i=1,\ldots ,k)$$ (1)

The function can be written as an extension:

$$f=f_{0}+\sum_{i}f_{i}+\sum_{i}\sum_{i>j}f_{ij}+\cdots +f_{12\ldots k}$$ (2)

Each of these terms is also square integrable over its domain of existence and is only a function of the corresponding factor in its subscript. Such as: *f*_*i*_ = *f*_*i*_(*Xi*), *f*_*ij*_ = *f*_*ij*_(*X*_*i*_, *X*_*j*_), If each term in the above expansion has a zero mean, that is: ∫*f*(*x*_*i*_)*dx*_*i*_ = 0, then all the items in the decomposition are one-to-one orthogonal, that is: *∫f*(*x*_*i*_)*f*(*x*_*j*_)*dx*_*i*_*dx*_*j*_ = 0. Therefore, these items can be expressed using the conditional expectation of the model output Y:

$$f_{0}=E(Y)$$ (3)

$$f_{i}=E(Y|X_{i})-E(Y)$$ (4)

$$f_{ij}=E(Y|X_{i},\;X_{j})-f_{i}-f_{j}-E(Y)$$ (5)

If the conditional expectation *E*(*Y*|*X*_*i*_) on *X*_*i*_ value has changed a lot, then *X*_*i*_ factor is important. Therefore, the variance of conditional expectation can be considered as a general term for sensitivity. The variances of the items in the above decomposition are the important measures being sought. In particular, *V*(*f*_*i*_(*X*_*i*_)) is *V*[*E*(*Y*|*Xi*)];When we divide this by the unconditional variance V(Y) we get the first order sensitivity index. That is:

$$S_{i}=\frac{V[E(Y|X_{i})]}{V(Y)}$$ (6)

It represents the main effect contribution of each input factor to the output variance.

Two factors are said to interact when their effects on Y cannot be expressed in terms of the sum of their individual effects. Interaction is an important feature of the model and the key to the Sobol method.

Further decomposition of (4) and (5) can be obtained as follows:

$$V_{i}=V(f_{i}(X_{i}))=V[E(Y|X_{i})]$$ (7)

$$V_{ij}=V(f_{ij}(X_{i},\;X_{j}))=V(E(Y|X_{i},\;X_{j}))-V(E(Y|X_{i}))-V(E(Y|X_{j}))$$ (8)

Where *V*(*E*(*Y*|*X*_*i*_, *X*_*j*_)) is the joint effect of (*X*_*i*_, *X*_*j*_) on Y, and *V*(*f*_*ij*_) is the joint effect of *X*_*i*_ and *X*_*j*_ minus the first-order effect of *X*_*i*_ and *X*_*j*_. *V*(*f*_*ij*_) is called a second-order or bidirectional effect [21].

*V*(*f*_*i*_) is simplified to *V*_*i*_, *V*(*f*_*ij*_) is simplified to *V*_*ij*_, and so on, Eq (2) can be written into the ANOVA-HDMR decomposition equation as follows:

$$V(Y)=\sum_{i}V_{i}+\sum_{i}\sum_{j>i}V_{ij}+\cdots +V_{12\ldots k}$$ (9)

Divide both sides by *V*(*Y*) to get:

$$\sum_{i}S_{i}+\sum_{i}\sum_{j>i}S_{ij}+\sum_{i}\sum_{j>i}\sum_{l>j}S_{ijl}+\cdots +S_{123\ldots k}=1$$ (10)

For factor *X*_*i*_, the total effect index refers to the total contribution of the factor to the change of model output, that is, the first-order effect of factor *X*_*i*_ plus all the higher-order effects generated by the interaction. The first-order effect of *X*_*i*_ is expressed by *S*_*i*_, and the total effect of *X*_*i*_ is expressed by $S_{T_{i}}$. The first-order effect calculation formula is (6), and the total effect calculation formula is:

$$S_{T_{i}}=\frac{E[V(Y|X_{∼i})]}{V(Y)}=1-\frac{V[E(Y|X_{∼i})]}{V(Y)}$$ (11)

When $S_{T_{i}}$ = 0 or *S*_*Ti*_≅0, *X*_*i*_ is a non-influence factor, i.e. any value of *X*_*i*_ within its value range will not significantly affect the value of the model output variance *V*(*Y*).

### 3.2 Sobol method calculation process

The number of samples is set as N (in this paper, it is set as 500). The larger the number of samples, the more accurate the results are [22]. The number of input variables is d (d = 6 in this paper). The general processing flow of Sobol method is as follows [23]:

1. The sample sampling method is generally based on Monte Carlo or its variants. Refer to the Sobol method [24, 25], and use Sobol’ quasi-random sequence to generate uniformly distributed (quasi) random numbers [22]. The realization of Sobolset function in Matlab, namely:
$$J_{n\times 2d}=[\begin{array}{ccccc} j_{1,1} & \cdots & j_{1,d} & \cdots & j_{1,2d}\\ \vdots & & \vdots & & \vdots \\ j_{n,1} & \cdots & j_{n,d} & \cdots & j_{n,2d} \end{array}]$$ (12)
2. Take the first d columns of matrix J as matrix A, and the remaining d columns as matrix B, so that two independent sample matrices of n points of the d-dimensional unit hypercube are given, namely:
$$A=[\begin{array}{ccc} j_{1,1} & \cdots & j_{1,d}\\ \vdots & & \vdots \\ j_{n,1} & \cdots & j_{n,d} \end{array}]$$ (13)
$$B=[\begin{array}{ccc} j_{1,d+1} & \cdots & j_{1,2d}\\ \vdots & & \vdots \\ j_{n,d+1} & \cdots & j_{n,2d} \end{array}]$$ (14)
  Then construct an n×d matrix *AB*^*i*^, so that the i-th column of *AB*^*i*^ is equivalent to the i-th column of matrix B, and the remaining columns are consistent with matrix A, namely:
$$AB^{i}=[\begin{array}{cccccc} j_{1,1} & \cdots & j_{1,i-1} & j_{1,d+i} & \cdots & j_{1,d}\\ \vdots & & \vdots & \vdots & & \vdots \\ j_{n,1} & \cdots & j_{n,i-1} & j_{n,d+i} & \cdots & j_{n,d} \end{array}]$$ (15)
  Where i = 1, 2, 3, …,
  So far, a total of (d+2) matrices of A, B, and *AB*^*i*^ have been constructed, and a sample of input parameters on the interval [0,1] of (d+2)×n group is obtained.
  Calculate the model output of all input values in the sample matrices A, B and *AB*^*i*^, and get 3 N-dimensional matrices.
$$y_{A}=f(A),y_{B}=f(B),y_{{AB}^{i}}=f({AB}^{i})$$ (16)
3. Calculate the sensitivity of each parameter according to the calculation formulas of the first-order influence index and total effect index above, namely:
$$S_{i}=\frac{V(E[Y|X_{i}])}{V(Y)}=\frac{y_{B}\cdot y_{{AB}^{i}}-f_{0}^{2}}{y_{B}\cdot y_{B}-f_{0}^{2}}=\frac{(1/N)\sum_{j=1}^{N}y_{B}^{\left(j\right)}y_{{AB}^{i}}^{\left(j\right)}-f_{0}^{2}}{(1/N)\sum_{j=1}^{N}{\left(y_{B}^{\left(j\right)}\right)}^{2}-f_{0}^{2}}$$ (17)
$$S_{T_{i}}=1-\frac{V[E(Y|X_{∼i})]}{V(Y)}=1-\frac{y_{A}\cdot y_{{AB}^{i}}-f_{0}^{2}}{y_{B}\cdot y_{B}-f_{0}^{2}}=1-\frac{(\frac{1}{N})\sum_{j=1}^{N}y_{A}^{\left(j\right)}y_{{AB}^{i}}^{\left(j\right)}-{f_{0}}^{2}}{(1/N)\sum_{j=1}^{N}{\left(y_{B}^{\left(j\right)}\right)}^{2}-{f_{0}}^{2}}$$ (18)
  $Where,\;f_{0}^{2}={\left(\frac{1}{N}\sum_{j=1}^{N}y_{B}^{\left(j\right)}\right)}^{2}$.

## 4. Sensitivity analysis and simulation of sensitive factor

### 4.1 Sensitivity analysis results and analysis

1)  Sensitivity Analysis of Coupled Models with the Same Interaction Radius

Sampling in Matlab, the number of samples is 500, and the parameters are input into the disease-information double-layer coupling network model to get the output result. We ran the model 4000 times in total, and the time step of the model was 50 time steps each time. Calculate the sensitivity and total effect index of each parameter section with the obtained running results in Matlab, and obtain the sensitivity indexes of each input parameter to the four model output variables $\rho_{A_{I}}、\rho_{N_{I}}、\rho_{S_{E}}、\rho_{R_{E}}$. The results are shown in Tables 2–5.

**Table 2:** Sensitivity index of each variable to $\rho_{A_{I}}$.

The input variable	*S*_*i*_	Sorting	The input variable	$S_{T_{i}}$	Sorting
***β***_***I***_	0.7702	1	*β*_*I*_	0.8965	1
***μ***_***I***_	0.1394	2	*μ*_*I*_	0.1961	2
***μ***_***E***_	-0.0312	6	*μ*_*E*_	0.053	4
***β***_***E***_	-0.0103	5	*β*_*E*_	0.0704	3
**ω**	0.0069	3	ω	-0.0191	6
***p***_***jump***_	-0.0004	4	*p*_*jump*_	0.0395	5

**Table 3:** Sensitivity index of each variable to $\rho_{N_{I}}$.

The input variable	*S*_*i*_	Sorting	The input variable	$S_{T_{i}}$	Sorting
***β***_***I***_	0.2353	1	*β*_*I*_	0.7203	1
***μ***_***I***_	0.2104	2	*μ*_*I*_	0.4704	2
***μ***_***E***_	-0.1193	6	*μ*_*E*_	0.3592	4
***β***_***E***_	-0.1031	5	*β*_*E*_	0.5584	3
***ω***	-0.0653	4	*ω*	0.1194	6
***p***_***jump***_	0.019	3	*p*_*jump*_	0.1536	5

**Table 4:** Sensitivity index of each variable to $\rho_{S_{E}}$.

The input variable	*S*_*i*_	Sorting	The input variable	$S_{T_{i}}$	Sorting
***β***_***I***_	-0.0263	5	*β*_*I*_	0.0295	5
***μ***_***I***_	-0.0259	6	*μ*_*I*_	0.0105	6
***μ***_***E***_	0.5582	1	*μ*_*E*_	0.6666	1
***β***_***E***_	0.1988	2	*β*_*E*_	0.2917	2
***ω***	-0.0049	4	Ω	0.0407	4
***p***_***jump***_	0.0334	3	*p*_*jump*_	0.1643	3

**Table 5:** Sensitivity index of each variable to $\rho_{R_{E}}$.

The input variable	*S*_*i*_	Sorting	The input variable	$S_{T_{i}}$	Sorting
***β***_***I***_	-0.009	5	*β*_*I*_	0.0276	5
***μ***_***I***_	-0.0154	6	*μ*_*I*_	0.0104	6
***μ***_***E***_	0.2719	2	*μ*_*E*_	0.3973	2
***β***_***E***_	0.4118	1	*β*_*E*_	0.5911	1
**ω**	0.0245	4	ω	0.0347	4
***p***_***jump***_	0.1087	3	*p*_*jump*_	0.1612	3

It can be seen from Table 2 that the most obvious factors affecting $\rho_{A_{I}}$ are information propagation rate *β*_*I*_ and information recovery rate *μ*_*I*_, while other factors have little effect on $\rho_{A_{I}}$.

It can be seen from Table 3 that the factors that have the most obvious effect on $\rho_{N_{I}}$ are information transmission rate *β*_*I*_ and information recovery rate *μ*_*I*_. The total effect index after each variable interacts with other variables is greater than the first-order effect index. Among them, the two factors of disease transmission rate *β*_*E*_ and disease recovery rate *μ*_*E*_ alone have a small impact on $\rho_{N_{I}}$, and the first-order impact index *S*_*i*_ is only -0.1031 and -0.1193, but they have a significant impact on $\rho_{N_{I}}$ after interacting with other factors. The total effect index $S_{T_{i}}$ is 0.5584 and 0.3592 respectively.

It can be seen from Table 4 that the most obvious factors affecting $\rho_{S_{E}}$ are disease transmission rate *β*_*E*_, disease recovery rate *μ*_*E*_. The total effect index of the probability of an individual moving across regions *p*_*jump*_ is greater than the first-order effect index, indicating that its interaction with other parameters has a greater impact on $\rho_{S_{E}}$, while other factors have little effect on $\rho_{S_{E}}$.

It can be seen from Table 5 that the most obvious factors affecting $\rho_{R_{E}}$ are the disease transmission rate *β*_*E*_, the disease recovery rate *μ*_*E*_ and the probability of individual movement across the region *p*_*jump*_, while other factors have little effect on $\rho_{R_{E}}$.

2) Sensitivity Analysis of Coupled Models with Heterogeneous Interaction Radius

Similar to the sensitivity analysis method of the previous model with the same interaction radius, we also performed a global sensitivity analysis on the improved coupling model of the heterogeneous interaction radius. The results are shown in Tables 6–9.

**Table 6:** Sensitivity index of each variable to $\rho_{A_{I}}$.

The input variable	*S*_*i*_	Sorting	The input variable	$S_{T_{i}}$	Sorting
***β***_***I***_	0.7241	1	*β*_*I*_	0.933	1
***μ***_***I***_	0.0589	3	*μ*_*I*_	0.2232	3
***μ***_***E***_	0.3512	2	*μ*_*E*_	0.4883	2
***β***_***E***_	-0.0188	4	*β*_*E*_	0.1869	4
**ω**	-0.0659	6	ω	0.0625	6
***p***_***jump***_	-0.0578	5	*p*_*jump*_	0.0976	5

**Table 7:** Sensitivity index of each variable to $\rho_{N_{I}}$.

The input variable	*S*_*i*_	Sorting	The input variable	$S_{T_{i}}$	Sorting
***β***_***I***_	0.3059	1	*β*_*I*_	0.6646	1
***μ***_***I***_	0.2658	2	*μ*_*I*_	0.3815	2
***μ***_***E***_	0.0143	5	*μ*_*E*_	0.1564	4
***β***_***E***_	0.0468	3	*β*_*E*_	0.3145	3
***ω***	-0.0402	3	*ω*	0.0391	5
***p***_***jump***_	0.0426	4	*p*_*jump*_	0.0358	6

**Table 8:** Sensitivity index of each variable to $\rho_{S_{E}}$.

The input variable	*S*_*i*_	Sorting	The input variable	$S_{T_{i}}$	Sorting
***β***_***I***_	-0.0457	6	*β*_*I*_	0.0565	4
***μ***_***I***_	-0.0267	5	*μ*_*I*_	0.0198	6
***μ***_***E***_	0.5802	1	*μ*_*E*_	0.6208	1
***β***_***E***_	0.2377	2	*β*_*E*_	0.2923	2
**ω**	-0.0198	4	ω	0.0495	5
***p***_***jump***_	0.0254	3	*p*_*jump*_	0.1256	3

**Table 9:** Sensitivity index of each variable to $\rho_{R_{E}}$.

The input variable	*S*_*i*_	Sorting	The input variable	$S_{T_{i}}$	Sorting
***β***_***I***_	-0.0529	6	*β*_*I*_	0.0595	4
***μ***_***I***_	-0.0155	5	*μ*_*I*_	0.0058	6
***μ***_***E***_	0.3319	2	*μ*_*E*_	0.3365	2
***β***_***E***_	0.4839	1	*β*_*E*_	0.554	1
**ω**	-0.0015	4	ω	0.0454	5
***p***_***jump***_	0.0835	3	*p*_*jump*_	0.1116	3

It can be seen from Table 6 that the most obvious factors affecting $\rho_{A_{I}}$ are information propagation rate *β*_*I*_ and information recovery rate *μ*_*E*_. The total effect of information recovery rate *μ*_*I*_ and disease transmission rate *β*_*E*_ on $\rho_{A_{I}}$ is significantly greater than the first-order effect, indicating that the impact on information dissemination becomes greater after interaction with other variables. In addition, in comparison with Table 2, it can be seen that the impact of disease on information is also more obvious in the coupled model of heterogeneous interaction radius.

It can be seen from Table 7 that the factors that have the most obvious effect on $\rho_{N_{I}}$ are information transmission rate *β*_*I*_ and information recovery rate *μ*_*I*_. Disease transmission rate *β*_*E*_ and disease recovery rate *μ*_*E*_ alone have little effect on $\rho_{N_{I}}$. The first-order impact index *S*_*i*_ is only 0.0468 and 0.0143, but after they interact with other factors, they have a significant impact on $\rho_{N_{I}}$, and the total effect index $S_{T_{i}}$ is 0.3145 and 0.1564 respectively.

It can be seen from Table 8 that the most obvious factors affecting $\rho_{S_{E}}$ are disease transmission rate *β*_*E*_, disease recovery rate *μ*_*E*_. The total effect index of the probability of an individual moving across regions *p*_*jump*_ is greater than the first-order effect index, indicating that its interaction with other parameters has a greater impact on $\rho_{S_{E}}$, while other factors have little effect on $\rho_{S_{E}}$.

It can be seen from Table 9 that the most obvious factors affecting $\rho_{R_{E}}$ are the disease transmission rate *β*_*E*_, the disease recovery rate *μ*_*E*_ and the probability of individual movement across the region *p*_*jump*_, while other factors have little effect on $\rho_{R_{E}}$.

### 4.2 Simulation results and analysis of sensitive factors

Based on the most influential parameter obtained by the above global sensitivity analysis, we select two values within the value range of this parameter to simulate and analyze the results. Each simulation result is the average result of 20 experiments.

Simulation results of the influence of different information dissemination rate *β*_*I*_ values on $\rho_{A_{I}}$

**Fig 2:**
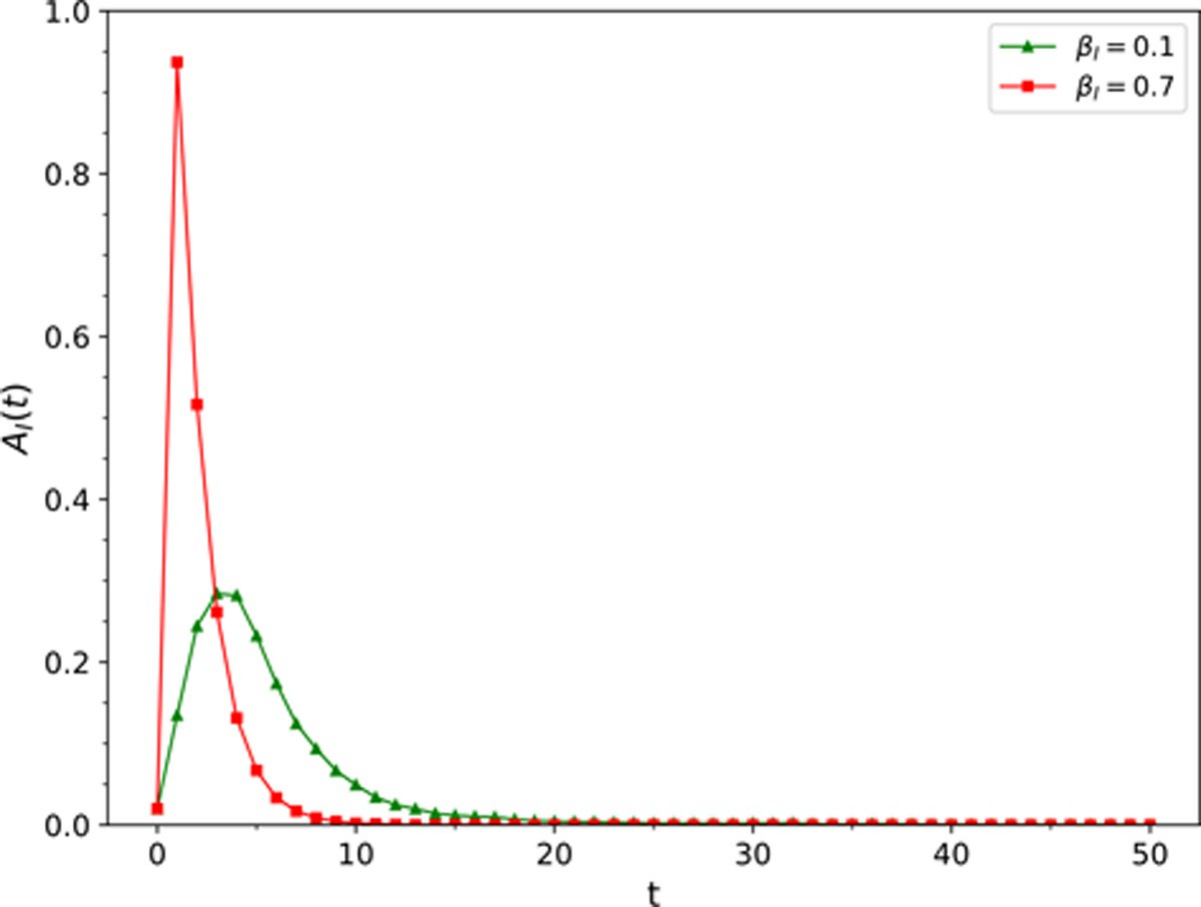
The influence of the information disseminatioin rate *β*_*I*_ on $\rho_{A_{I}}$.

The fixed parameter values were *μ*_*I*_ = 0.5, *β*_*E*_ = 0.6, *μ*_*E*_ = 0.1, *r* = 1, *v* = 0.03, *ω* = 0.2, and *p*_*jump*_ = 0.01, and the values of *β*_*I*_ were 0.1 and 0.7. The change of the density of nodes in the information dissemination state in the information layer over time is presented in Fig 2. It was found that the information dissemination rate increased from 0.1 to 0.7, the peak density of nodes in state *A*_*I*_ of the information layer increased from 28.4% to 93.7%.

Simulation results of the influence of different information dissemination rate *β*_*I*_ values on $\rho_{N_{I}}$

**Fig 3:**
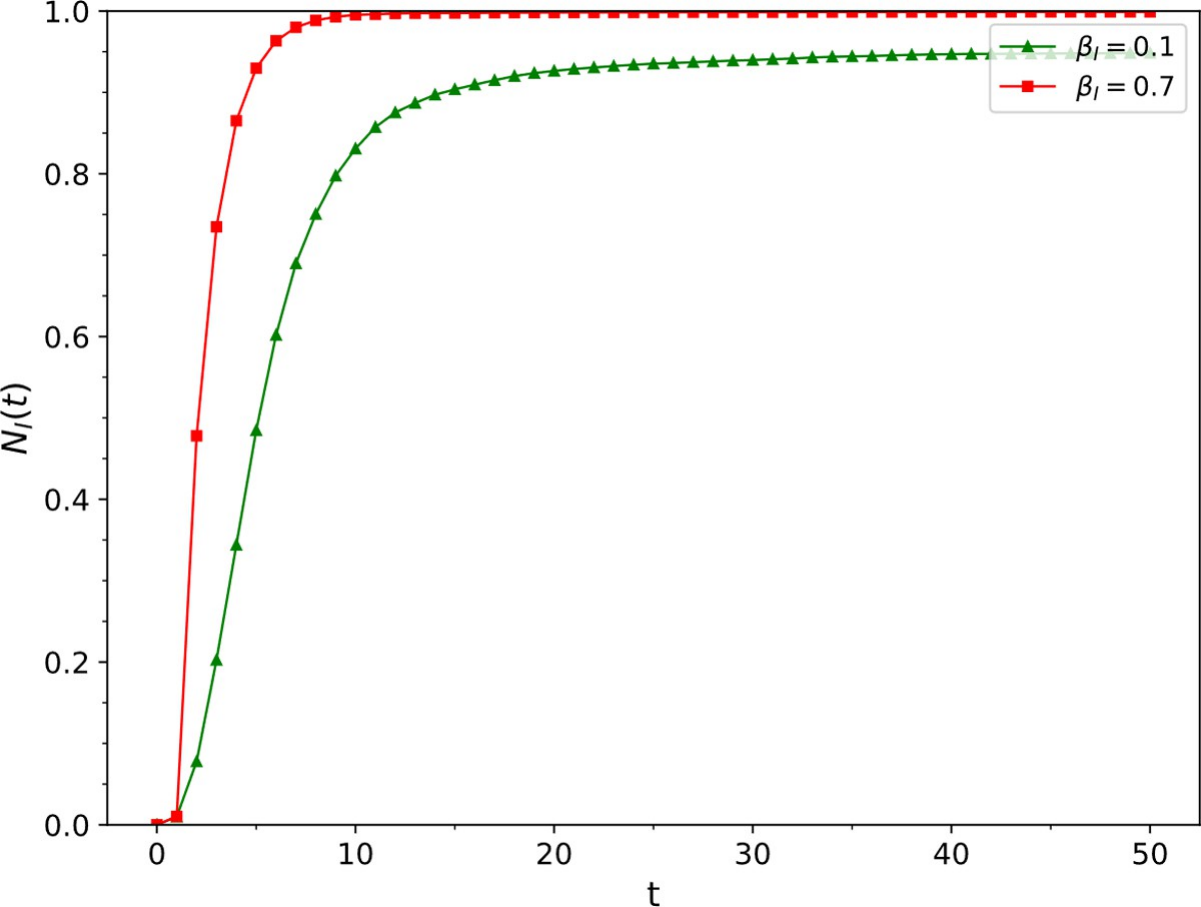
The influence of the information disseminatioin rate *β*_*I*_ on $\rho_{N_{I}}$.

The fixed parameter values were *μ*_*I*_ = 0.5, *β*_*E*_ = 0.6, *μ*_*E*_ = 0.1, *r* = 1, *v* = 0.03, *ω* = 0.2, and *p*_*jump*_ = 0.01, and the values of *β*_*I*_ were 0.1 and 0.7. The change of the density of nodes in the information recovery state in the information layer over time is presented in Fig 3. It was found that the information dissemination rate increased from 0.1 to 0.7, and the information dissemination scale increased from 94% to 99%.

**Fig 4:**
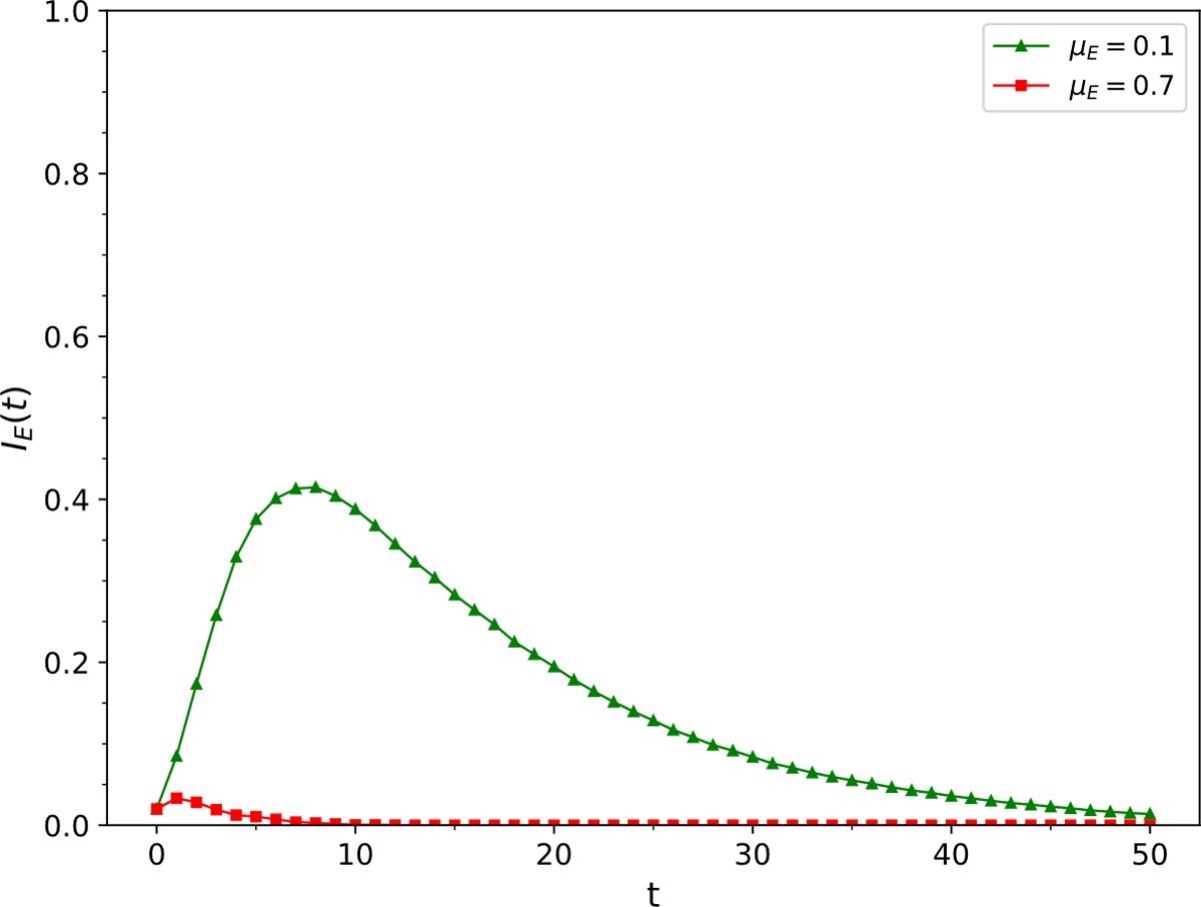
The influence of epidemic recovery rate *μ*_*E*_ values on $\rho_{S_{E}}$.

Simulation results of the influence of different epidemic recovery rate *μ*_*E*_ values on $\rho_{S_{E}}$

we conducted a simulation by changing the epidemic recovery rate *μ*_*E*_ while keeping all the other parameters fixed as follows: *β*_*I*_ = 0.1, *μ*_*I*_ = 0.5, *β*_*E*_ = 0.6, *r* = 1, *v* = 0.03, *ω* = 0.2, and *p*_*jump*_ = 0.01, The values of *μ*_*E*_ were 0.1 and 0.7. The change of the density of nodes in the epidemic infection state in the epidemic layer over time is presented in Fig 4. It was found that the epidemic recovery rate increased from 0.1 to 0.7, the peak density of infected nodes in the epidemic layer decreased from 41.5% to 3.3%.

Simulation results of the influence of different epidemic transmission rate *β*_*E*_ values on $\rho_{R_{E}}$

**Fig 5:**
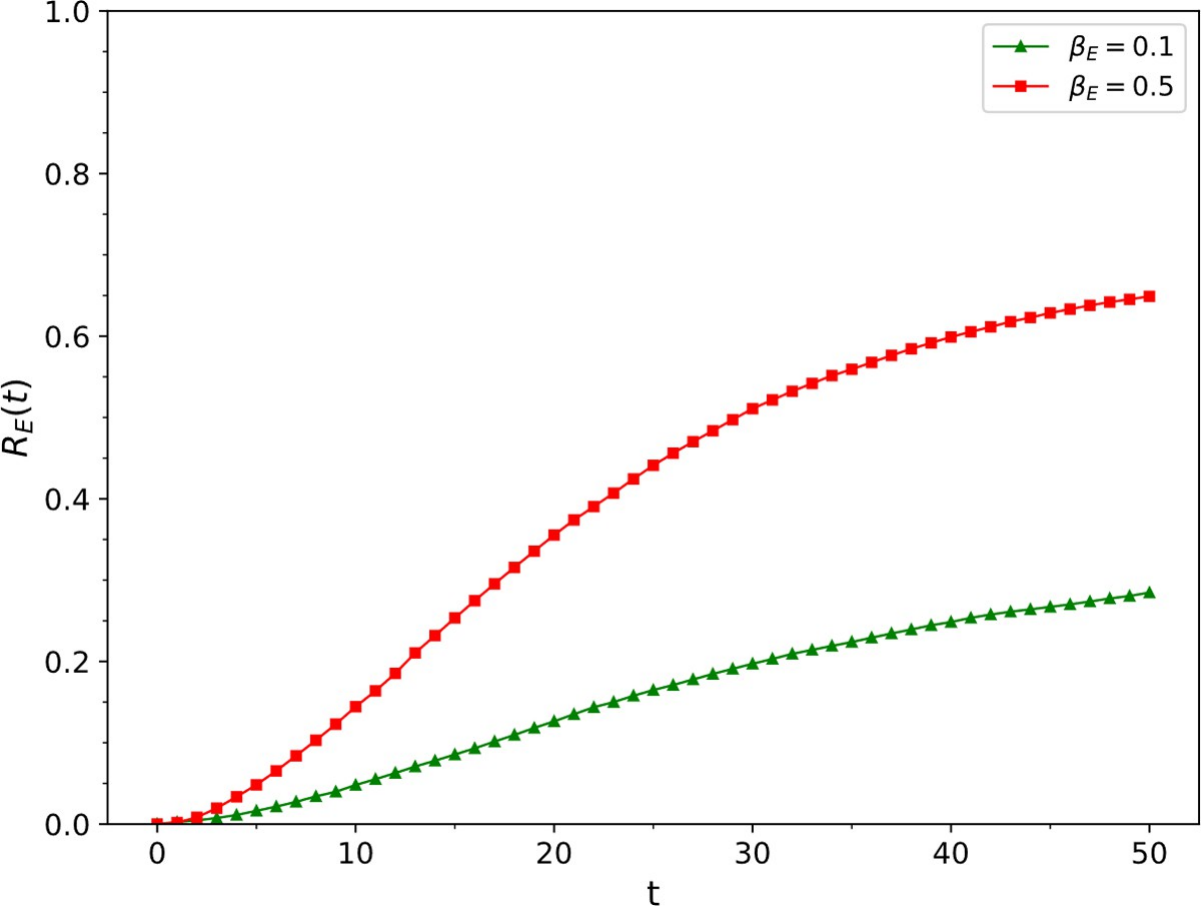
The influence of epidemic transmission rate *β*_*E*_ values on $\rho_{R_{E}}$.

We conducted this simulation by changing the epidemic transmission rate *β*_*E*_ and fixing the other parameters. The fixed parameter values were *β*_*I*_ = 0.1, *μ*_*I*_ = 0.5, *μ*_*E*_ = 0.1, *r* = 1, *v* = 0.03, *ω* = 0.2, and *p*_*jump*_ = 0.01, the values of *β*_*E*_ were 0.1 and 0.5. The change of the density of nodes in the epidemic recovery state in the epidemic layer over time is presented in Fig 5. It was found that the epidemic transmission rate increased from 0.1 to 0.5, the scale of the epidemic spread increased from 28% to 64.5%.

## 5. Conclusion

The qualitative and quantitative analysis of the input and output of complex models and systems by the sensitivity analysis method is conducive to the diagnosis of the model structure, the identification of model parameters and the application of the model. In this paper, the Sobol method based on variance is used to analyze the global sensitivity of the disease information coupling dynamics model of the same interaction radius and heterogeneous interaction radius. There are 6 model parameters, namely: information transmission rate, information recovery rate, disease transmission rate, disease recovery rate, the probability of moving across regions and the probability of taking preventive actions. There are four output variables of the model: the peak density of nodes in the *A*_*I*_ state of the information layer, density of nodes in the *N*_*I*_ state when the information layer dissemination approaches the end (the scale of information dissemination), the peak density of nodes in the *S*_*E*_ state of the epidemic layer, and the density of nodes in the *R*_*E*_ state when the spread of the disease layer approaches the end (the scale of disease spread).

Sensitivity analysis results show that: (1) The parameters that have the most obvious impact on the peak density of nodes in the *A*_*I*_ state and the scale of information dissemination are the information dissemination rate *β*_*I*_ and the information recovery rate *μ*_*I*_. Therefore, if the dissemination of information is to be controlled, measures need to be taken to control these two parameters; (2) The parameters that have the most obvious impact on the peak density of nodes in the *S*_*E*_ state of the disease layer and the scale of disease transmission are disease transmission rate *β*_*E*_, disease recovery rate *μ*_*E*_, and the probability of individual movement across regions *p*_*jump*_. Measures need to be taken to control these three parameters to control the spread of the disease; (3) The parameter value range will significantly affect the calculation results of parameter sensitivity. It can be seen from our previous research results that in a certain parameter the value interval has a more obvious influence on the model output, while other value intervals have a smaller influence on the model output. Studies by other scholars have confirmed this point [26].

Although this article attempts to analyze the global sensitivity of the parameters of the disease-information double-layer coupled network model, there are still some shortcomings in the research of this article. Only the Sobol method is used and no other global sensitivity analysis methods were applied. In the next step, we will use other methods to analyze the sensitivity of the model, and compare the analysis results of the various methods. In addition, we will further divide the parameter value range to explore the influence of different parameter value ranges on parameter sensitivity. The control research of multivariate system can also provide reference for our future research [27, 28].
